# Watching the Barn Door Open—Timing CML Stem Cell Allografts

**DOI:** 10.1002/ajh.27722

**Published:** 2025-05-20

**Authors:** Jeffrey H. Lipton

**Affiliations:** ^1^ Princess Margaret Cancer Centre University of Toronto Toronto Ontario Canada

**Keywords:** CML, optimal timing, stem cell allografting

1

*The timing of your decision is just in important as the decision you make*.

John C. Maxwell

The management of CML has evolved over the last quarter century or so, transforming this once fatal disease into one that is curable or at least controllable [1]. With the routine use of tyrosine kinase inhibitor (TKI) therapy, life expectancy is near normal compared to age‐matched controls [2] although quality of life (QOL) may not be normal [3, 4]. Survival has not really improved beyond what was seen with the first generation of these drugs, but newer drugs have helped with issues of resistance and intolerance, making the need to use more aggressive options a much less frequent event.

In the pre‐TKI era, the only option for possible “cure” with the rare exception of interferon therapy, was an allogeneic bone marrow, now stem cell transplant [5, 6]. This, however, was a reality if a patient was young, usually under the age of 40 years, had a matched sibling, had reasonable health, was near a transplant program, and had reimbursement for this costly procedure. In the western world, where the median age of CML diagnosis is in the mid‐60s, or the developing world, where the procedure was not technically available or reimbursed, it can be seen that the majority of patients were left with palliative management only. If this was all that was available, major developments in allografting have occurred, which have changed the landscape. Patients can now be transplanted into their eighth decade, co‐morbidities can often be managed, reduced intensity conditioning is less acutely toxic, better graft‐versus‐host disease (GVHD) prophylaxis and therapy are available, costs are down, and more centers are available. But most significantly, with the availability of a larger donor pool—alternate related donors, unrelated donor registries, and haploidentical donors—allografting could be available to more people [7, 8]. The converse of this is…so what. With the appearance of imatinib and subsequent TKIs, the need for allografting almost disappeared, and along with it, the memory that allografting still exists and may be appropriate in a small number of cases.

In parallel, advancements in TKI development have yielded newer drugs that in many cases can deal with resistance or intolerance of earlier drugs and for some patients with co‐morbidities, may be safer and without the problem of adverse events or side effects [9, 10]. This has reduced but not eliminated the need to consider an allograft. On the other hand, more frequent chronic problems can sometimes be replaced with more serious issues or, in cases of the newest drugs, unknown long‐term issues, if any. Risk/benefit ratios become important, and the use of a potentially “riskier” drug can be justified with more advanced CML, after careful thought, discussion, and consent.

This commentary will not deal in general with the indications for allografting. I want to use the term “timing” to reflect not the disease state when to transplant [11], but when to do it once the decision becomes clear. The indications have been well reviewed [5, 6] and other than the few cases where a newer drug can overcome resistance or intolerance, remain the same. Issues like advanced disease, clonal progression or disease progression, uncontrolled multi‐drug resistance or persistent hematological toxicity remain the arguments for considering it. What I would like to consider here is the timing of an allograft, or to paraphrase Goldilocks in nursery rhymes—not too soon, not too late, but just right!

Historically pre‐TKI, it has been shown that allografting early after diagnosis, within one to 2 years, is optimal for the best outcome [12]. Even transplanting after failed alpha‐interferon was very successful long term [13]. Transplant at a later time or with more advanced disease had inferior results [14]. There was concern that the early benefits would be lost by the delay that results from attempting therapy with TKI before moving on to allografting. For the most part, this has been shown to be a non‐issue even after second generation drugs if the transplant was done in chronic phase [15, 16, 17]. This has been shown to be true even with expanded donor options and transplant approaches [18, 19].

CML has traditionally been treated by two separated groups—the conventional CML treaters and the transplanters. On rare occasions, an individual may do both, which may be an advantage when allografting enters the picture. In this day and age, the former is by far the larger group and may consist of qualified hematologists, oncologists, and generalists. Depending on the size of the CML practice, this comes with differing degrees of expertise, comfort, and perspective. For example, a practice may have a huge benign component, a solid tumor component, and a lesser malignant hematology component made up of many diseases, with CML being uncommon. Given that so many CML patients have no major issues, it is understandable that some of the intricacies of an uncommon disease may not be obvious to this group. The concern that is raised by the transplanter is usually based on why referral is so late, with obvious worsening of disease or general health. Recommendations from the NCCN [20] or soon to be updated ELN [21], or local, which take available resources into consideration, should be consulted.

I want to start with my impression of the reasons for delayed or missed referral for allografting. I expect that the most common is that the role of allografting for CML has not been on the radar for years or decades. Unlike autografting for myeloma or allografting for acute leukemia, which are now built into management algorithms, allografting for CML is not. This may be due to CML education, whether academically based or marketing driven, that primarily deals with TKIs and competes with educational programs for other diseases that are more common. The second reason would most likely be due to a concern about the toxicity of an allograft. Much has changed over the decades, and transplants are safer—not completely safe, but safer than they were decades ago. But then TKI therapy is not completely safe [22]. The third may very well be the wrong impression of who is eligible for a transplant—age, disease status, donor availability, co‐morbidities. Unless someone is in a center where allografts are being done for any disease, there may exist unknowns or old information, and a lot has changed. The fourth is more concerning. The attitude that this is something to consider when there is nothing else available is ancient history, and this will be discussed below. The last could be due to a reimbursement or transplant availability issue, which is now outside the bounds of a medical decision.

The reasons for refusal to transplant vary but usually are based on not meeting the eligibility criteria for a “standard” transplant. Disease may have progressed, for example, uncontrolled blast crisis, the rare case now where there is no donor identified, poor co‐morbid health, patient unwilling to consent to the transplant or appropriate follow‐up. Some centers may offer “experimental” or “study” transplants which will consider patients otherwise ineligible.

Why is delaying an allograft risky for either a higher risk of failure of the transplant or even for being able to get a transplant? There are a number of criteria on this list. They include the disease now not having a likely chance of benefiting or a high risk of relapse, worsening candidate health makes the risk of treatment‐related mortality or morbidity higher, the donor no longer being available because of health reasons or concerns about their own risks or donor disappearance from a registry, either temporarily or permanently, waiting lists at some centers, and changes in reimbursement coverage. Once eligible, always eligible is not a reality. There is a quality window here.

How to proceed then with getting the appropriate patient to a timely allograft? There are a number of branches in this decision road, and I will try to identify my approach. The first is simple. Is this patient a transplant candidate? In deciding this, the patient has exhausted all other therapies that can yield a durable response, not just all other available therapies. In the absence of resistance directed by specific mutations that might respond to another TKI, or intolerance that may be specific drug related, now is the time. Flipping between second or third generation drugs for the sake of allograft avoidance or delay puts the patient at risk. If the need for an allograft is inevitable, then why wait? If there is still insistence on trying another drug, then rare positive results should be available in 2–3 months, and if they are not obvious, move on.

A delay in a patient who may have another medical condition that can be corrected to reduce allograft risk could be considered. An example might be cardiac stent placement. The same might be true for the best donor, so long as the delay does not affect the patient.

Start the process. Above all, if unsure, a referral to a transplant program is prudent. Initiation of family member identification and HLA‐typing, unrelated donor search if it is done outside of the transplant center, and referral to other services for patient “buffing” should be done sooner rather than later.

Allografts after TKI therapy are still very effective if done on time. Logic should tell us, that at best a delay will not improve outcome and in fact may yield an inferior result. So why wait? In the words of George Bernard Shaw—“Nothing is worth doing unless the consequences may be serious.” A missed successful transplant opportunity is what I would call serious.

## Disclaimer

J.L. is responsible for all aspects of this commentary.

## Ethics Statement

No patient material is included, so no Review Board was necessary.

## Conflicts of Interest

The author declares no conflicts of interest.

## Data Availability

There are no data stored and available.
